# MicroRNA dataset of normal and *Nosema ceranae*-infected midguts of *Apis cerana cerana* workers

**DOI:** 10.1016/j.dib.2019.104518

**Published:** 2019-09-14

**Authors:** Yu Du, Dingding Zhou, Huazhi Chen, Cuiling Xiong, Yanzhen Zheng, Dafu Chen, Rui Guo

**Affiliations:** College of Bee Science, Fujian Agriculture and Forestry University, Fuzhou, 350002, China

**Keywords:** *Apis cerana cerana*, *Nosema ceranae*, Midgut, MicroRNA, Transcriptome

## Abstract

*Nosema ceranae* is a widespread fungal pathogen of honeybees, which is infective to all castes in the colony, including queens, drones and workers. Nosemosis caused by *N. ceranae* poses a big challenge for apiculture all over the world. Here, midguts of normal and *N. ceranae*-infected *Apis cerana cerana* workers at 7 and 10 days post infection were sequenced utilizing small RNA sequencing (sRNA-seq) technology. Totally, more than 150.54 Mb raw reads were produced in this article, and over 144.26 Mb high-quality clean reads with a mean ratio of 95.83% were obtained after strict filtering and quality control. For more insight please see “Comparative identification of microRNAs in *Apis cerana cerana* workers' midguts responding to *Nosema ceranae* invasion” (Chen et al., 2019). Raw data are available in NCBI Sequence Read Archive (SRA) database under the BioProject number PRJNA487111. Our data can be used for investigating differentially expressed microRNAs (miRNAs) and piRNAs and their regulatory roles engaged in *A. c. cerana* response to *N. ceranae* infection, and for offering potential candidates for uncovering the molecular mechanisms regulating eastern honeybee-microsporidian interactions.

**Table:** Specifications table

Subject	*Biology*
Specific subject area	*Transcriptomics*
Type of data	*Table, Figure*
How data were acquired	*Illumina MiSeq*
Data format	*Raw sequences (FASTQ) and processed data (FASTA)*
Experimental factors	*Normal and Nosema ceranae-infected midguts of Apis cerana cerana workers*
Experimental features	*Midgut samples in control groups were harvested from A. c. cerana workers inoculated with sterile sucrose solution, while midgut samples in treatment groups were harvested from workers inoculated with sterile sucrose solution containing N. ceranae spores. Total RNA of control and N. ceranae-infected groups were extracted followed by small RNA library construction and next-generation sequencing using the single-end strategy.*
Data source location	*College of Bee Science, Fujian Agriculture and Forestry University, Fuzhou, China*
Data accessibility	*Raw data of small RNA-seq (sRNA-seq) are available on Sequence Read Archive (SRA) database and connected to BioProject PRJNA487111*
Related research article	*D.F. Chen, Y. Du, H.Z. Chen, Y.C. Fan, X. X. Fan, Z.W. Zhu, J. Wang, C.L. Xiong, Y.Z. Zheng, C.S. Hou, Q.Y. Diao, R. Guo. Comparative identification of microRNAs in Apis cerana cerana workers' midguts in response to Nosema ceranae invasion. Insects (2019), 10, 258, doi:**10.3390/insects10090258*.

**Table:** 

**Value of the data** • The datasets offer comprehensive information associated with small RNAs including miRNAs and piRNAs in normal and *N. ceranae*-infected *A. c. cerana* workers. • Our data provide a valuable genetic resource and potential candidates for further investigation of the regulatory roles of miRNAs involve in N. ceranae-response of *A. c. cerana*. • This data is beneficial for deciphering the molecular mechanisms regulating the eastern honeybee-microsporidian interactions.

## 1 Data

**Fig. 1:**
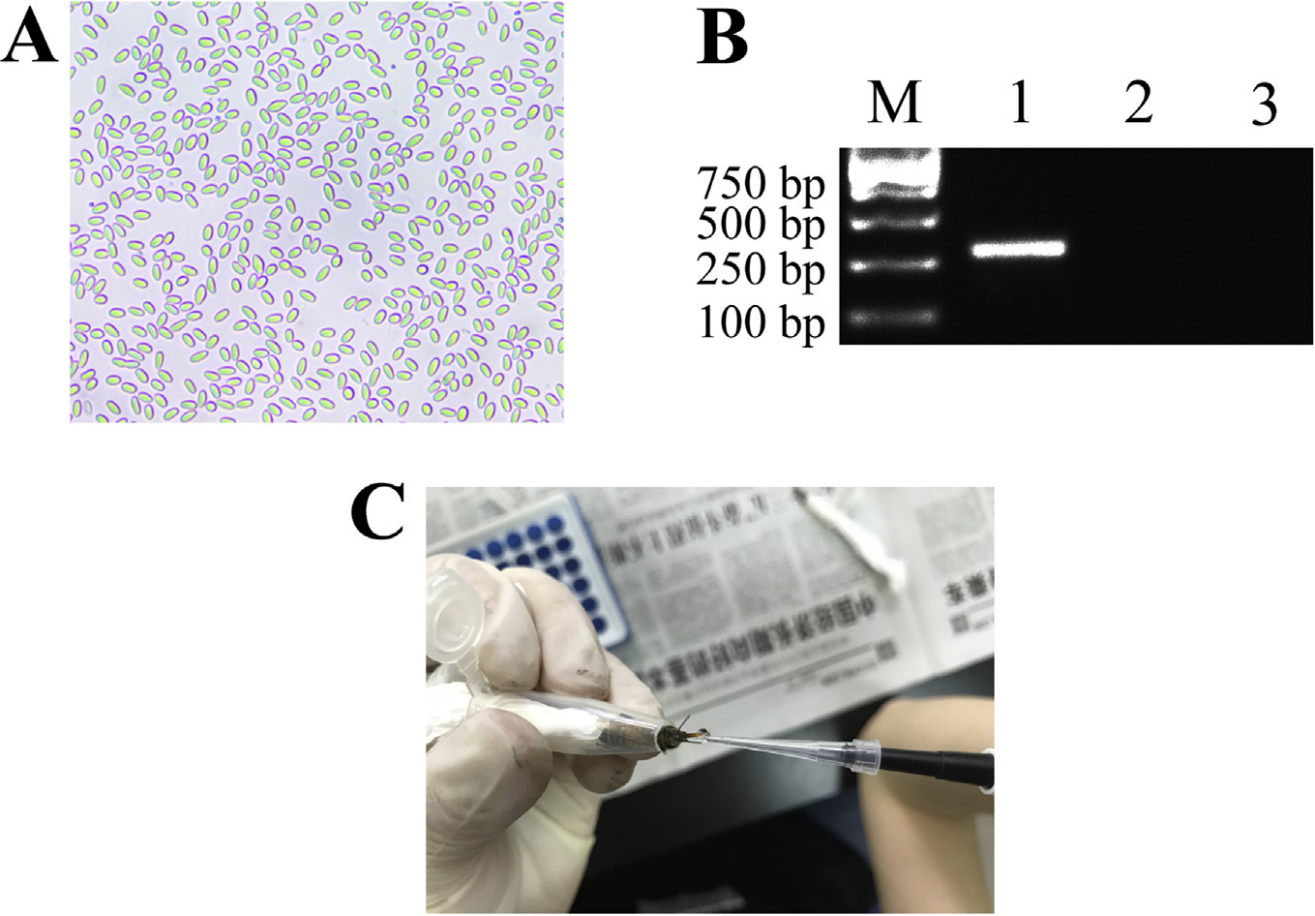
PCR validation of purified *N. ceranae* spores and artificial inoculation of *A. c. cerana* worker. **A)** Microscopic observation of *N. ceranae* spores purified via Percoll discontinuous density gradient centrifugation (400 times magnification). **B)** Agarose gel electrophoresis of PCR products from the purified spores. Lane 1 and Lane 2 display amplification products using specific primers of *N. ceranae* and *Nosema apis*, respectively. Lane 3: Sterile water (negative control). Lane M: DNA marker. **C)** Artificial inoculation of each worker with sterile sucrose containing or without *N. ceranae* spores.

**Fig. 2:**
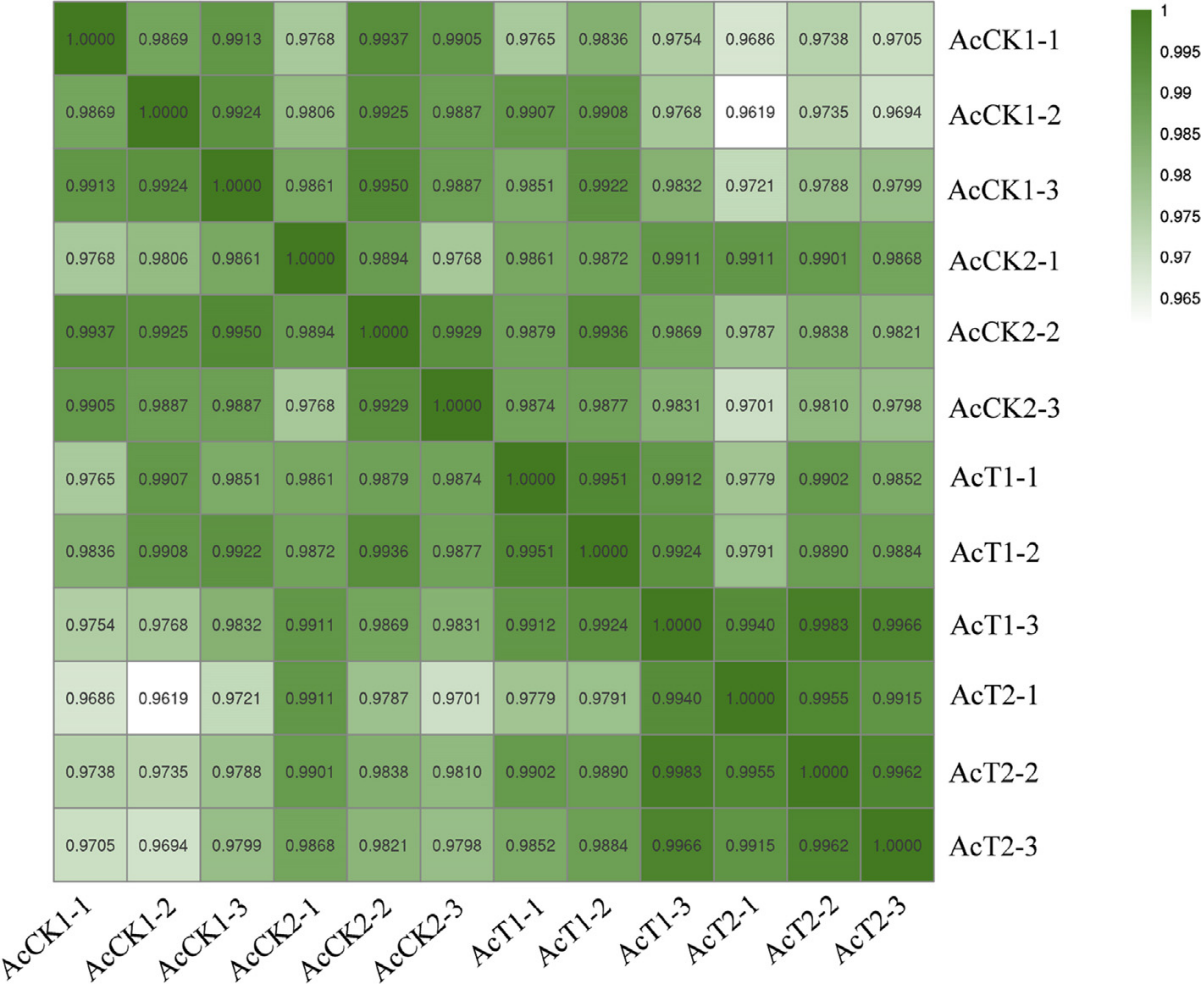
Pearson correlation coefficients between different biological replicas within each normal and *N. ceranae*-infected group.

*N. ceranae* spores (Fig. 1A) were purified with Percoll discontinuous density gradient centrifugation, followed by validation with specific primers and agrose gel electrophoresis (Fig. 1B). After being starved for 2 h, each worker of *A. c. cerana* was artificially inoculated with 50% sucrose solution containing *N. ceranae* spores (Fig. 1C). The shared miRNA profile is from normal and *N. ceranae*-infected midguts of *A. c. cerana* workers [1]. On average, more than 12.54 Mb raw reads in each group were yielded from sRNA-seq, and over 12.02 Mb (95.83%) clean reads were gained after strict filtering and quality control (Table 1). Additionally, Pearson correlation coefficients between different biological replicas within each control and *N. ceranae*-infected group were above 0.9768 and 0.9912, respectively (Fig. 2) [1]. In total, 14 differentially expressed miRNAs (DEmiRNAs) were observed in midgut at 7 days post inoculation (dpi) with *N. ceranae* (AcT1) compared with corresponding normal midgut (AcCK1), including eight up-regulated and six down-regulated miRNAs (Table 2); while 12 miRNA with differential expressions were detected in midgut at 10 dpi with *N. ceranae* (AcT2) compared with corresponding normal midgut (AcCK2), including nine up-regulated and three down-regulated ones (Table 3). The raw data were deposited in the Sequence Read Archive (SRA) database (http://www.ncbi.nlm.nih.gov/sra/) and connected to BioProject PRJNA487111.

**Table 1:** Quality control of sRNA-seq datasets.

Sample	Raw reads	Clean reads
AcCK1-1	12757706	12049987 (94.45%)
AcCK1-2	12794543	12122624 (94.75%)
AcCK1-3	11328883	10727931 (94.70%)
AcCK2-1	17161911	16292122 (94.93%)
AcCK2-2	11666305	11306117 (96.91%)
AcCK2-3	11757223	11328016 (96.35%)
AcT1-1	11213906	10923950 (97.41%)
AcT1-2	14549245	13778004 (94.70%)
AcT1-3	11029767	10800311 (97.92%)
AcT2-1	13263930	12775381 (96.32%)
AcT2-2	13688082	13150156 (96.07%)
AcT2-3	12936357	12467737 (96.38%)

**Table 2:** Summary of DEmiRNAs in AcCK1 vs AcT1 comparison group.

miRNA	Sequence	Length	TPM in AcCK1	TPM in AcT1	Log_2_(Fold change)	*p* value	Mark
miR-676-y	GCTGTCCTAAGGTAGATGA	19	0.01	80.42	12.97	2.96E-05	Up
miR-60-y	ACATGTTCTGGTTGAAGA	18	0.01	18.70	10.87	1.51E-05	Up
miR-2965-y	AGGACTGCTACAGAGAGCA	19	0.01	17.17	10.75	3.91E-05	Up
miR-8462-x	ATTAATTTGATAAGTTATA	19	0.01	14.42	10.49	0.000242	Up
miR-6717-x	GCGATGTGGGGACGGAGA	18	0.01	12.06	10.24	2.14E-07	Up
miR-6313-y	TGCTGTGAAGTTTTGATT	18	0.01	5.97	9.22	2.21E-06	Up
miR-3726-x	GAGTGGTGGATGCCAGCGTT	20	0.01	5.17	9.02	0.000162	Up
miR-252-y	CTGCTGCTCAAGTGCTTATCA	21	11.17	27.02	1.27	0.025931	Up
miR-980-y	AAGCTGCCTTTTGAAGGGCAACA	23	39.09	18.85	−1.05	0.038812	Down
miR-598-y	GTCGTCGTCGTCATCGTCA	19	2.41	0.01	−7.92	9.16E-05	Down
miR-1-x	CCGTGCTTCCTTACTTCCCATA	22	3.34	0.01	−8.38	2.82E-07	Down
miR-965-x	GGGGAAAGGTTATAGCGATTATG	23	3.64	0.01	−8.51	4.12E-07	Down
miR-4635-y	GAAGTCGGAACCCGCTAAG	19	5.68	0.01	−9.15	4.11E-05	Down
miR-9204-x	CTGGGATGAAATGTGGGT	18	6.02	0.01	−9.23	0.00087	Down

**Table 3:** Summary of DEmiRNAs in AcCK2 vs AcT2 comparison group.

miRNA	Sequence	Length	TPM in AcCK2	TPM in AcT2	Log_2_(Fold change)	*p* value	Mark
miR-676-y	GCTGTCCTAAGGTAGATGA	19	0.01	410.36	15.32	4.02E-06	Up
miR-60-y	ACATGTTCTGGTTGAAGA	18	0.01	205.03	14.32	0.000287	Up
miR-194-y	CAGTGGGGCGGTTGTTAT	18	0.01	67.96	12.73	6.20E-06	Up
miR-6313-y	TGCTGTGAAGTTTTGATT	18	0.01	34.23	11.74	0.000177	Up
miR-2965-y	AGGACTGCTACAGAGAGCA	19	0.01	29.17	11.51	1.18E-06	Up
miR-8462-x	ATTAATTTGATAAGTTATA	19	0.01	18.29	10.84	4.28E-05	Up
miR-7338-y	TTTAGCTGGTTTGTCAAGA	19	0.01	9.56	9.90	2.31E-05	Up
miR-3654-y	GCGACTGGAAAAGCTGAA	18	0.01	6.91	9.43	2.64E-06	Up
miR-3720-x	TACGGTGATGAGTTTAAA	18	24.49	63.42	1.37	0.023317	Up
novel-m0019-5p	AGTCTCGATCGAGACATGTGA	21	8.37	0.01	−9.71	1.58E-06	Down
novel-m0003-3p	TGGTGATATGTGTATATACTGATT	24	8.92	0.01	−9.80	8.91E-05	Down
miR-92-x	TTGGGCGGGGTGTCCGTGC	19	31.95	0.01	−11.64	0.001613	Down

## 2 Experimental design, materials, and methods

### 2.1 Honeybee midgut sample preparation

Frames of a sealed brood comb from a healthy colony of *A. c. cerana* were kept in an incubator at 34 ± 2 °C to offer newly emerged *Nosema*-free workers. Workers 24 h after eclosion were used for artificial inoculation, following the previously developed standard method [2]. In brief, each worker in *N. ceranae*-treated group was fed with 5 μL of a 50% sucrose (w/w in water) solution containing 1 × 10^6^ *N. ceranae* spores [3], while each worker in control group was fed with 5 μL of a 50% sucrose solution without *N. ceranae* spores. There were three cages (30 workers per cage) for each *N. ceranae*-treated group and three cages (30 workers per cage) for each control group. Midguts of nine workers from each cage in the *N. ceranae*-treated and control groups were respectively collected at 7 dpi and 10 dpi and immediately pooled, frozen in liquid nitrogen, and stored at −80 °C until deep sequencing.

### 2.2 Small RNA library construction and next-generation sequencing

Small RNA libraries were constructed according to the general protocol [1]. Briefly, total RNA of each midgut sample in *N. ceranae*-treated and control groups were extracted using TRIzol Reagent followed by removal of DNA contaminants; only values of 28S/18S ≥ 0.7 and RIN ≥7.0 were considered qualified for the subsequent small RNA library construction. RNA molecules in the size range of 18–30 nt were enriched by agarose gel electrophoresis and then ligated with 3′ and 5’ RNA adaptors, followed by enrichment of fragments with adaptors on both ends by PCR after reverse transcription; the subsequent cDNAs were enriched by 3.5% AGE to isolate the expected size (140–160 bp) fractions; deep sequencing of the 12 cDNA libraries were conducted on Illumina MiSeq platform using the single-end strategy. The libraries were as follows: AcCK1-1, AcCK1-2 and AcCK1-3 as replicate libraries for normal midguts at 7 dpi with sucrose solution; AcT1-1, AcT1-2 and AcT1-3 as replicate libraries for midguts at 7 dpi with sucrose solution containing *N. ceranae* spores; AcCK2-1, AcCK2-2 and AcCK2-3 as replicate libraries for normal midguts at 10 dpi with sucrose solution; AcT2-1, AcT2-2 and AcT2-3 as replicate libraries for midguts at 10 dpi with sucrose solution containing *N. ceranae* spores.

All sRNA sequencing data produced in our study are available in NCBI SRA database under BioProject number: PRJNA487111.

### 2.3 Quality control and sequence analysis

The raw data generated from the platform were pre-processed to exclude low-quality reads (length < 20 nt and ambiguous N), 5′ adapter, 3′ adapter and poly(A) sequences, then the obtained clean reads were aligned against NCBI GeneBank and Rfam databases to remove noncoding RNA such as rRNA, scRNA, snoRNA, snRNA and tRNA, followed by comparison with exons and introns in the *A. cerana* genome (assembly ACSNU-2.0) to classify mRNA degradation products and the repeat associate miRNA sequences. All the downstream analyses were carried out using the clean reads with high quality.

Bowtie (v 1.1.0) [4] was used to align the filtered sequences against miRBase 21.0 by allowing at most two mismatches outside of the seed region, and small RNAs that matched exist miRNAs of other animal species in miRBase were identified as known miRNAs. The sequences that did not match known miRNAs were used to predict potentially novel miRNA candidates using RNAfold software [5]. Only sequences with typical Stem-loop hairpins, mature length distributed between 18 nt and 26 nt and free energy lower than −20 kcal/mol were considered as potential novel miRNAs. The suffixes "-x" and "-y" mean a certain miRNA deriving from the processing of the 5′ and 3’ arms of its precursor, respectively; while the suffix "-z" means a certain miRNA with unknown processing direction.

The miRNA expression levels in each sample were normalized to the total number of sequence tags per million (TPM) following the formula: normalized expression = mapped read count/total reads × 10^6^. The differential expression of miRNAs in each comparison group was analyzed using the DEGseq R package [6]. The criteria of *p* value＜0.05 and |log_2_(Fold change)|＞1 were set as the threshold for statistically significant differential expression, and *p* values were adjusted using *q* value.
